# Sex-specific association of sleep duration with subclinical indicators of metabolic diseases among asymptomatic adults

**DOI:** 10.1186/s12944-022-01626-w

**Published:** 2022-01-23

**Authors:** Lili Huang, Zichong Long, Gang Xu, Yiting Chen, Rong Li, Yanlin Wang, Shenghui Li

**Affiliations:** 1 School of Public Health, Shanghai Jiao Tong University School of Medicine, 227 South Chongqing Road, 200025 Shanghai, China; 2 International Peace Maternity & Child Health Hospital, Shanghai Jiao Tong University School of Medicine, 910 Hengshan Road, 200030 Shanghai, China; 3 MOE-Shanghai Key Laboratory of Children’s Environmental Health, Shanghai Jiao Tong University School of Medicine, 1665 Kongjiang Road, 200092 Shanghai, China

**Keywords:** Metabolic health, Sleep duration, TG/HDL-C, TyG, LAP, Sex-specific association

## Abstract

**Background:**

Accumulating evidence suggests sleep duration may be involved in metabolic regulation. However, studies regarding the association with the early stage of the metabolic disease are limited, and the findings were inconsistent.

**Methods:**

A study among 4922 asymptomatic adults was conducted based on a Chinese national survey in 2009. The early stage of metabolic diseases was evaluated using three proxies: triglyceride to high-density lipoprotein cholesterol ratio (TG/HDL-C), the product of triglyceride and fasting glucose (TyG), and lipid accumulation product (LAP). Multivariable linear and logistic regression models were used to explore the associations of sleep duration with the three indicators.

**Results:**

The linear regression models revealed that, among females, sleep duration <7 h per day, compared with 7-9 h, was associated with an increased value of LAP and TyG by 25.232% (95%CI: 10.738%, 41.623%) and 0.104 (95%CI: 0.024, 0.185), respectively, in the crude model. The effects were attenuated but remained significant for LAP (11.405%; 95%CI: 1.613%, 22.262%). Similarly, the logistic regression models further found that sleep duration <7 h per day could increase the risk of elevated LAP (OR: 1.725, 95CI%:1.042, 2.856) after adjusting for multiple covariates. By contrast, no associations were found among males.

**Conclusions:**

Short sleep duration was associated with subclinical indicators of metabolic diseases, and females were more susceptible to the association.

**Supplementary Information:**

The online version contains supplementary material available at 10.1186/s12944-022-01626-w.

## Background

Sleep is critically important in an individual’s physiological function maintenance, making it a potential modifiable candidate in health promotion and disease prevention. As suggested by an increasing number of studies, sleep may be involved in metabolic rhythm and glucose metabolism [1, 2]. Sleep duration is the core parameter of sleep, while it is easily disturbed by various factors, including physical and psychosocial factors, etc. [3, 4]. The health risk caused by abnormal sleep duration has been a public concern [5, 6]. Epidemiological data have reported the associations of sleep duration with clinical metabolic diseases such as diabetes [7], hypertension [8], and cardiovascular diseases (CVD) [9]. However, the short or long sleep duration may also be the consequence of comorbidities, such as depression, which may confound the relationship between sleep duration and outcomes. Therefore, it is essential to explore the relationship between sleep duration and early-stage indicators among asymptomatic participants who are less likely to be affected by drug use or comorbidities.

Clinical metabolic-related diseases, such as diabetes, hypertension, and CVD, are the leading cause of death among middle-aged and older populations worldwide. Dyslipidemia is one of the most indisputable risk factors [10, 11]. Systematic reviews and meta-analyses based on clinical trials suggest that managing dyslipidemia could directly reduce the CVD risk [12]. Therefore, it is a prerequisite to determine the appropriate evaluation index of dyslipidemia, representing the subclinical state of metabolic diseases. In recent years, several novel indexes, such as triglyceride (TG) to high-density lipoprotein cholesterol (HDL-C) ratio (TG/HDL-C) [13, 14], the product of triglyceride and fasting glucose (TyG) [15], and lipid accumulation product (LAP) [16], were found to be good predictors of subsequent metabolic diseases. They have been recommended as early clinical surrogate markers of metabolic diseases. Data from Mexico reported that TG/HDL≥3.0 could strongly predict prediabetes among women aged 40-60 years [17]. A Korean study proposed 8.81 as the optimal cut-off value of TyG to screen early-stage metabolic diseases [18]. Similarly, higher than the 75th percentile of LAP value could effectively predict metabolic diseases, as suggested by a study in China [19].

To date, quite a few studies estimated the association of sleep with these novel indexes; the existing data mainly focused on obstructive sleep apnoea (OSA) [20–23], and the association between sleep duration and these subclinical indicators is almost a blank. To date, only one study conducted in Korea found that participants who slept >8 h and 7-8 h could increase the risk of higher LAP [24]. Therefore, this study aims to explore the association of sleep duration with subclinical indicators of metabolic diseases, including TG/HDL-C, TyG, and LAP among asymptomatic participants. Considering the notable sex differences in metabolic disease risk [25], the analyses were conducted stratified by sex.

## Methods

### Study design

The current study used data from the China Health and Nutrition Survey (CHNS), a national survey conducted from1989. The available data, including both sleep duration and clinical detection, was only in 2009. This work was approved by the institutional review committees of the National Institute of Nutrition and Food Safety, the Chinese Center for Disease Control and Prevention, the University of North Carolina at Chapel Hill, and the China-Japan Friendship Hospital, Ministry of Health before the recruitment of participants. A written consent form was signed for each participant before participating in this study.

### Measurements of sleep duration and the subclinical indicators of metabolic disease

Sleep duration was measured by the question “How many hours each day do you usually sleep, including daytime and nighttime (hours)?”. According to the joint consensus statement of the American Academy of Sleep Medicine and Sleep Research Society, sleep duration of 7-8 h per day is recommended for adults, and sleep duration <7 h or ≥9 h may increase the risk of adverse health conditions [5]. In the current study, sleep duration was classified into three groups: <7 h, 7-9 h, and ≥9 h.

Fasting blood samples were collected by trained technicians and transferred to the national central lab for detection. Fast plasma glucose (FPG) was measured by the GOD-PAP method (Randox Laboratories Ltd, UK). TG and HDL-C were tested using GPO-PAP and the enzymatic method (Kyowa, Japan), respectively. Hypersensitive C-reactive protein (hs-CRP) was determined by the immunoturbidimetric method (Denka Seiken, Japan reagents). Inflammation was defined as hs-CRP >2 mg/L based on its relationship with a higher risk of CVD in China [26].

The subclinical indicators were calculated referring to the following formula: TG/HDL-C: TG (mg/dL)/HDL-C (mg/dL); LAP: [waist circumference (WC) - 65] × [TG (mmol/L)] for males or [WC - 58] × [TG (mmol/L)] for females; TyG: ln [TG (mg/dL) ×FBG (mg/dL)/2] [14]. Currently, there were no recommended criteria to cut the three indicators into normal vs. elevated groups, and most studies used the upper quantile value to define elevated value [19, 27]. The participants were apparently healthy in this study, so the 90th quantile was chosen. The elevated TG/HDL-C, LAP, and TyG were defined as >6.30, >70.03, and >9.43 for males, respectively; and >4.58, >65.48. and >9.25 for females, respectively.

### Covariates

Height, weight, and WC, and blood pressure were measured according to standard protocol, and detailed methods have been introduced in previous studies [14]. Blood pressure was measured by trained technicians in triplicate, and the averaged value was used in analyses. Body mass index (BMI) was calculated as weight (kilograms) divided by height (meters) square. Leisure-time physical activity (PA) per week was calculated by multiplying the time spent in each activity (hours) by metabolic equivalents for that activity. Thus, the total leisure-time PA was expressed as the sum of metabolic equivalent hours for all leisure time activities [28]. Sedentary time per day was calculated based on screen exposure time (video games, joining chat rooms, surfing the internet, watching television, etc.) and other sedentary activities. As suggested by published literature, multiple factors were associated with dyslipidemia [29, 30]. Regarding the variable selection method in multivariate analysis, those variables were identified as covariates if they could result in >10% change of the estimated coefficients in the bivariate analysis [31]. In the present study, age (<40, 40-59, ≥60 years), BMI, serum uric acid (UA), region (north vs. south), area of the city (urban vs. rural), education (less than primary, middle-high school, tech and more), leisure PA (tertiles), sedentary time (tertiles), ever smoking (yes vs. no), alcohol drinking (yes vs. no), and inflammation (yes vs. no) were included.

### Statistical analysis

The description of participants’ demographic characteristics, sleep duration, and subclinical indicators of metabolic diseases were stratified by sex. Kolmogorov-Smirnov test was used to assess the normality of data distribution. TyG showed normal distribution, and TG/HDL-C and LAP were transformed into a logarithmic scale to normalize their distribution. The sex difference of characteristics was assessed using Student’s test, Kruskal-Wallis test, or chi-square tests where appropriate. The distributions of subclinical indicators of metabolic diseases were analyzed according to the sleep duration groups; the differences were assessed using the Kruskal-Wallis test or Student’s test.

When TG/HDL-C, TyG, and LAP were analyzed as continuous variables, multivariable linear regression models were used to explore the associations of sleep duration with the three indicators. The estimated effect was transformed into percent change using the formula: (exp(B)-1) *100 for TG/HDL-C and LAP to interpret the results. Further, multivariable logistic models were used to estimate the odds ratio of sleep duration on elevated indicators. Specifically, only sleep duration was included in the crude model. Then age, region, area of the city, education, leisure PA, sedentary time, ever smoking, alcohol drinking, BMI were furtherly included in the adjusted model 1. Finally, serum UA and inflammation were additionally adjusted in model 2.

The analyses in this study were performed by IBM SPSS Statistics (version 24.0, IBM Corp.); *P*< 0.05 (two-sided) was considered statistically significant.

## Results

### The enrollment of participants

In this study, participants older than 18 years were enrolled in the analysis. Of 10,286 adults, 79 participants with pregnancy, 1100 with diabetes by self-report or by clinical detection (FPG ≥7mmol/L or Hemoglobin A1c ≥6.5%), 90 with heart disease, stroke, or other cardiovascular diseases, and 1722 with hypertension (diagnosed by clinicians or taking anti-hypertension drugs) were excluded. Further, 134 with missing or extreme data on sleep duration, 2239 with missing or extreme data on TG, HDL-C, serum UA, hs-CRP, BMI, blood pressure, or WC were excluded. Finally, 4922 participants remained in the final analysis (males: 2194; females: 2728) (Figure S1). Compared with the participants who entered the final analysis, those excluded tended to be older; had higher BMI, WC, systolic blood pressure, and diastolic blood pressure; were more likely to smoke, drink alcohol, and live in urban and the north area (Table S1).

### The characteristics of the analyzed participants

Table 1 summarized the participants’ characteristics stratified by sex (males: 44.6%). The mean [standard deviation, (SD)] age in males was 47.78 (14.77) years, which was slightly older than female [45.65 (13.69) years] (*P*=0.001). The mean sleep duration was similar between sex [7.98 (1.14) hours in males, 7.98 (1.11) hours in females]. Compared with females, males had higher WC, TG, serum UA, hs-CRP, and blood pressure levels but lower HDL-C concentration (*P*<0.001). The value of TG/HDL-C, LAP, and TyG in males was also higher than in females (*P*<0.001).

**Table 1:** Characteristics of participants stratified by sex, mean (SD) or n (%)

	Males (*n*=2194)	Females (*n*=2728)	*P*
**Characteristics**			
Age (years)	47.78 (14.77)	45.65 (13.69)	0.001
<40	671 (30.6)	931 (34.1)	0.001
40-59	1055 (48.1)	1368 (50.1)	
≥60	468 (21.3)	429 (15.7)	
BMI (kg/m^2^)	22.62 (3.07)	22.67 (3.12)	
WC (cm)	82.15 (9.19)	78.69 (9.28)	<0.001
UA (mg/dl)	5.27 (0.98)	4.04 (0.91)	<0.001
TC (mmol/L)	4.66 (0.91)	4.70 (0.96)	
TG (mmol/L)	1.35 (0.85)	1.28 (0.85)	0.003
LDL-C (mmol/L)	2.89 (0.92)	2.90 (0.90)	
HDL-C (mmol/L)	1.43 (0.46)	1.50 (0.37)	<0.001
Abnormal TC	552 (25.2)	761 (27.9)	0.031
Abnormal TG	525 (23.9)	543 (19.9)	<0.001
Abnormal LDL-C	541 (24.7)	708 (26.0)	
Abnormal HDL-C	215 (9.8)	130 (4.8)	<0.001
Dyslipidemia	1046 (47.7)	1197 (43.9)	0.008
Glucose (mmol/l)	5.03 (0.63)	4.99 (0.57)	0.022
Hs-CRP (mg/L)	2.36 (6.03)	1.88 (10.63)	<0.001
Inflammation			
Yes	423 (19.3)	442 (16.2)	<0.001
No	1771 (80.7)	2286 (83.8)	
SBP (mmHg)	119.65 (11.84)	114.14 (11.59)	<0.001
DBP (mmHg)	78.07 (7.93)	74.37 (7.69)	<0.001
Region			
North	1191 (54.3)	1498 (54.9)	
South	1003 (45.7)	1230 (45.1)	
Area of city			
Urban	650 (29.6)	860 (31.5)	
Rural	1544 (70.4)	1868 (68.5)	
Education			
Less than primary	720 (32.8)	1195 (43.8)	<0.001
Middle-high school	1184 (54.0)	1198 (43.9)	
Tech and more	290 (13.2)	335 (12.3)	
Income (yuan)			
Low	743 (33.9)	912 (33.4)	
Medium	728 (33.2)	850 (31.2)	
High	723 (33.0)	966 (35.4)	
**Life styles**			
Leisure PA/week (MET)	138.52 (117.53)	138.23 (103.35)	0.021
Sedentary time (hours)	3.03 (2.48)	2.78 (2.13)	0.003
Low	614 (28.0)	782 (28.7)	
Medium	874 (39.8)	901 (33.0)	
High	706 (32.2)	1045 (38.3)	
Ever smoked			
Yes	1380 (62.9)	95 (3.5)	<0.001
No	813 (37.1)	2632 (96.5)	
Drank alcohol			
Yes	1309 (59.7)	252 (9.2)	<0.001
No	885 (40.3)	2475 (90.8)	
**Sleep and subclinical indicators**			
Sleep duration (hours)	7.98 (1.14)	7.98 (1.11)	
<7	183 (8.3)	211 (7.7)	
7-9	1517 (69.1)	1919 (70.3)	
≥9	494 (22.5)	598 (21.9)	
TG/HDL-C	2.47 (2.05)	2.18 (2.01)	<0.001
LAP	25.13 (25.13)	28.24 (25.73)	<0.001
TyG	8.43 (0.58)	8.37 (0.57)	<0.001

*BMI* body mass index, *WC* waist circumference, *TC* total cholesterol, *TG* triglyceride, *LDL-C* low-density lipoprotein cholesterol, *HDL-C* high-density lipoprotein cholesterol, *Hs-CRP* hypersensitive C-reactive protein, *UA* uric acid, *SBP* systolic blood pressure, *DBP* diastolic blood pressure, *PA* physical activity, *MET* metabolic equivalent, *LAP* lipid accumulation product, *TyG* triglyceride glucose

### The distributions of TG/HDL-C, LAP, and TyG based on sleep duration

Table 2 presented the distributions of TG/HDL-C, LAP, and TyG based on sleep duration groups. All three indicators did not show significant differences among sleep duration groups in males. In females, the differences were statistically significant except for TG/HDL-C. The highest value of LAP was observed in the group whose sleep duration was <7 h [median: 25.11 (interquartile range: 13.30, 45.12)], and the lowest value was found in those who slept ≥9 h [19.65 (11.44, 35.15)]. The highest value of TyG was also observed in participants whose sleep duration <7 h [8.42(8.05, 8.84)].

**Table 2:** The distributions of TG/HDL-C, LAP and TyG stratified by sleep duration groups

	n	TG/HDL	LAP	TyG
Males		Median (Interquartile range)		
<7 h	183	1.77 (1.10, 2.73)	16.24 (7.93, 33.20)	8.28 (7.97, 8.81)
7-9 h	1517	1.86 (1.14, 3.06)	18.40 (9.12, 33.06)	8.40 (8.02, 8.83)
≥9 h	494	1.92 (1.28, 3.10)	16.37 (8.25, 31.47)	8.40 (8.03, 8.82)
* P*		0.220	0.279	0.445
Females				
<7 h	211	1.78 (1.07, 3.10)	25.11 (13.30, 45.12)	8.42 (8.05, 8.84)
7-9 h	1919	1.64 (1.08, 2.57)	20.88 (11.22, 35.40)	8.35 (7.98, 8.73)
≥9 h	598	1.69 (1.03, 2.53)	19.65 (11.44, 35.15)	8.32 (7.95, 8.74)
* P*		0.465	0.003	0.020

TG/HDL-C: triglyceride (TG) to high-density lipoprotein cholesterol ratio (HDL-C); LAP: lipid accumulation product; TyG: triglyceride glucose

### The associations of sleep duration with TG/HDL-C, LAP, and TyG

As shown in Table 3, compared with those who slept 7-9 h, the female participants whose sleep duration <7 h increased 25.232% (95%CI: 10.738%, 41.623%) of LAP and 0.104 (95%CI: 0.024, 0.185) of TyG, respectively, in the crude model. After adjusting for multiple covariates, the estimated effects were attenuated but still significant for LAP (11.405%; 95%CI: 1.613%, 22.262%). By contrast, associations were not established among males.

**Table 3:** The association of sleep duration with TG/HDL-C, LAP and TyG, stratified by sex

	Crude model	Adjusted model 1	Adjusted model 2
	Percent change (95%CI)	Percent change (95%CI)	Percent change (95%CI)
**Males**			
LnTG/HDL-C^a^			
<7 h	-6.574 (-16.054, 3.873)	0.904 (-8.607, 11.516)	0.602 (-8.607, 10.849)
7-9 h	0 (Reference)	0 (Reference)	0 (Reference)
≥9 h	3.458 (-3.536, 10.960)	5.022 (-1.587, 12.187)	4.498 (-1.980, 11.293)
LnLAP^a^			
<7 h	-5.351 (-18.861, 10.407)	2.737 (-8.607, 15.488)	2.634 (-8.515, 15.142)
7-9 h	0 (Reference)	0 (Reference)	0 (Reference)
≥9 h	-7.411 (-16.389, 2.429)	-0.200 (-7.596, 7.788)	-0.698 (-7.965, 7.144)
TyG^b^			
<7 h	-0.064 (-0.154, 0.025)	-0.019 (-0.104, 0.066)	-0.022 (-0.104, 0.060)
7-9 h	0 (Reference)	0 (Reference)	0 (Reference)
≥9 h	-0.011 (-0.070, 0.048)	0.002 (-0.053, 0.058)	-0.003 (-0.056, 0.051)
**Females**			
LnTG/HDL-C^a^			
<7 h	6.823 (-2.664, 17.234)	2.840 (-6.012, 12.412)	1.410 (-6.947, 10.517)
7-9 h	0 (Reference)	0 (Reference)	0 (Reference)
≥9 h	-1.587 (-7.318, 4.603)	-0.299 (-5.824, 5.654)	0 (-5.446, 5.654)
LnLAP^a^			
<7 h	25.232 (10.738, 41.623)	12.975 (2.634, 24.234)	11.405 (1.613, 22.262)
7-9 h	0 (Reference)	0 (Reference)	0 (Reference)
≥9 h	-1.980 (-9.516, 6.077)	2.327 (-3.729, 8.872)	2.634 (-3.343, 8.981)
TyG^b^			
<7 h	0.104 (0.024, 0.185)	0.054 (-0.023, 0.130)	0.040 (-0.033, 0.113)
7-9 h	0 (Reference)	0 (Reference)	0 (Reference)
≥9 h	-0.021 (-0.074, 0.031)	-0.018 (-0.067, 0.031)	-0.015 (-0.062, 0.031)

TG/HDL-C: the ratio of triglyceride (TG) to high-density lipoprotein cholesterol (HDL-C); LAP: lipid accumulation product; TyG: triglyceride glucose

^a^ the estimated value were transformed into: (exp(estimated value)-1) *100 as percent change; ^b^ the estimated value was expressed as unit change

Adjusted model 1: adjusted for age, region, area of city, education, leisure PA, sedentary time, ever smoked, drank alcohol, BMI

Adjusted model 1: additionally adjusted for UA, inflammation

### The association of sleep duration with elevated TG/HDL-C, LAP, and TyG

Table 4 presented the associations between sleep duration and elevated TG/HDL-C, LAP, and TyG, stratified by sex. Consistent with linear regression models, the associations between sleep duration and elevated indicators were not established in males. While among females, compared with the group who slept 7 to 9 h, the group who slept <7 h had the increased risk of elevated LAP (OR: 1.921, 95%CI: 1.254, 2.942) and elevated TyG (OR: 1.656, 95%CI: 1.055, 2.599), respectively; however, sleep duration≥ 9 h decreased the risk of elevated TG/HDL, in the crude model. After adjusting for multiple covariates, only the association between sleep duration <7 h and elevated LAP was still significant though the effect was attenuated (OR: 1.725, 95%CI: 1.042, 2.856).

**Table 4:** The association of sleep duration with elevated TG/HDL-C, LAP, and TyG, stratified by sex

	Crude model	Adjusted model 1	Adjusted model 2
	OR (95%CI)	OR (95%CI)	OR (95%CI)
**Male**			
Elevated TG/HDL-C			
<7 h	0.790 (0.468, 1.334)	0.930 (0.534, 1.619)	0.927 (0.528, 1.628)
7-9 h	1.000 (Reference)	1.000 (Reference)	1.000 (Reference)
≥9 h	0.941 (0.681, 1.301)	0.943 (0.671, 1.325)	0.904 (0.637, 1.282)
Elevated LAP			
<7 h	0.527 (0.241, 1.151)	0.533 (0.227, 1.251)	0.544 (0.231, 1.282)
7-9 h	1.000 (Reference)	1.000 (Reference)	1.000 (Reference)
≥9 h	0.913 (0.607, 1.373)	1.004 (0.640, 1.576)	0.943 (0.596, 1.491)
Elevated TyG			
<7 h	0.717 (0.398, 1.292)	0.827 (0.448, 1.527)	0.829 (0.445, 1.547)
7-9 h	1.000 (Reference)	1.000 (Reference)	1.000 (Reference)
≥9 h	0.980 (0.694, 1.384)	0.985 (0.688, 1.412)	0.958 (0.662, 1.385)
**Female**			
Elevated TG/HDL-C			
<7 h	1.214 (0.752, 1.960)	1.148 (0.699, 1.886)	1.094 (0.651, 1.838)
7-9 h	1.000 (Reference)	1.000 (Reference)	1.000 (Reference)
≥9 h	0.662 (0.452, 0.971)	0.719 (0.486, 1.063)	0.733 (0.491, 1.096)
Elevated LAP			
<7 h	1.921 (1.254, 2.942)	1.763 (1.092, 2.847)	1.725 (1.042, 2.856)
7-9 h	1.000 (Reference)	1.000 (Reference)	1.000 (Reference)
≥9 h	0.864 (0.602, 1.241)	0.983 (0.663, 1.458)	0.986 (0.657, 1.479)
Elevated TyG			
<7 h	1.656 (1.055, 2.599)	1.571 (0.978, 2.521)	1.535 (0.930, 2.534)
7-9 h	1.000 (Reference)	1.000 (Reference)	1.000 (Reference)
≥9 h	1.002 (0.708, 1.420)	1.089 (0.758, 1.563)	1.131 (0.779, 1.644)

TG/HDL-C: the ratio of triglyceride (TG) to high-density lipoprotein cholesterol (HDL-C); LAP: lipid accumulation product; TyG: triglyceride glucose

OR: odds ratio; CI: confidence interval

Adjusted model 1: adjusted for age, region, area of city, education, leisure PA, sedentary time, ever smoked, drank alcohol, BMI

Adjusted model 1: additionally adjusted for UA, inflammation

## Discussion

In the present study, the sex-specific associations between sleep duration and subclinical indicators of metabolic disease, including TG/HDL-C, LAP, and TyG, were estimated among asymptomatic adults. The relationship between short sleep duration and LAP was still significant among females, even adjustment for multiple covariates. The findings extended the attention to the relationship between sleep duration and early stage of metabolic disease, highlighting the potentiality of sleep intervention in preventing and controlling metabolic disease. However, the sex difference is worthy of in-depth study.

So far, sparse data have evaluated the associations between sleep and novel subclinical indicators, most of which focused on OSA. In a case-control study conducted in non-diabetic and non-obese Europeans, the TyG levels were significantly higher in participants with OSA after adjustment for covariates [22]. Only a Korean study evaluated the associations of sleep duration with the novel subclinical indicators. It revealed that compared with those who slept <6 h, 7-8 h and >8 h of sleep duration were associated with an increased LAP [24]. In the current study, 7-9 h was the reference, and only sleep duration <7 h was associated with higher LAP. Existed evidence have revealed the associations of sleep duration with simple lipid and glucose metabolism indicators, such as WC [32], HDL-C [33], and TG [34, 35]. However, the conclusions were, to a large extent, controversial. By contrast, TG/HDL-C is obtained from the ratio of TG to HDL-C, showing a stronger correlation with insulin resistance than individual lipid profiles [14]. LAP combines WC and TG, reflecting physiological changes in lipid overaccumulation [36]; therefore, it has been regarded as the stronger predictor of a metabolic symptom than the traditional lipid accumulation index [37, 38]. TyG comes from TG and FPG and has a stronger predictive value for diabetes in normal glucose patients than FPG [39]. Nevertheless, in this study, TG/HDL and TyG were relatively susceptible to other factors, and their relationship with sleep duration was no longer significant after adjustment for covariates. To date, only several studies estimated the association between sleep duration and these indicators; more studies are needed to enrich the evidence.

The current findings established the association between short sleep duration (<7 h) and increased subclinical indicators of metabolic diseases, especially LAP among females, in linear and logistic regression models. Therefore, this study provides an epidemiological basis for linking short sleep duration to a higher risk of subsequent metabolic diseases. Indeed, these findings were consistent with previous studies regarding sleep duration and clinical metabolic diseases [40, 41]. A previous study using the same database as in this study also revealed that participants who slept ≤7 h, compared with 8 h, had a higher risk of hypertension [40]. Additionally, a meta-analysis revealed that short sleep duration was associated with higher risks of diabetes mellitus (OR:1.37, 95%CI:1.22-1.53), hypertension (OR:1.17, 95%CI:1.09-1.26), and CVD (OR: 1.16, 95%CI:1.10-1.23) [41].

The potential mechanisms of short sleep duration with higher subclinical indicators are still unclear. Evidence has demonstrated that sleep loss may induce leptin decrease [42] and ghrelin increase [43], both of which could result in obesity [44]. Similarly, short sleep duration may increase serum uric acid, which has been revealed as a risk factor for metabolic diseases [45, 46]. Moreover, short sleep duration could trigger a metabolic regulation-related inflammatory reaction [47, 48]. Sleep deprivation was also associated with endothelial dysfunction [49], leading to metabolic diseases [50]. Additionally, sleep and metabolism are intertwined physiologically, through which biological arousal may directly affect metabolic systems in the hypothalamus [51].

Notably, the effects of short sleep duration on subclinical indicators were more likely to be found in females. There is notable sex divergence in the risk of metabolic disease, which may be related to sex-specific hormonal and behavioral differences [52, 53]. In addition, the evidence indicated that sleep duration deficiency might be associated with an elevated inflammatory level in females but not in males [54]. The sex differences need to be confirmed in further studies.

### Study strengths and limitations

The major strengths of the current study lie in the use of novel indicators that represent the early stages of metabolic disease and only asymptomatic adults selected as the study sample. Some limitations should be noted. Firstly, the cross-sectional study design limited the power to establish a causal association. Secondly, sleep duration was measured by a self-reported question rather than an objective method (such as actigraphy); fortunately, evidence has reported a good correlation between the objective measurement and the subjective reported data [55]. Meanwhile, only sleep duration was observed, while other parameters, such as sleep quality and OSA, were unavailable, which were also associated with metabolic disease [56, 57]. Thirdly, there are no recommended cut-offs for elevated TG/HDL-C, LAP, and TyG; the present study used the 90th percentile, which may not have the optimal sensitivity and specificity. In addition, although participants with the metabolic disease were excluded, information was unavailable on whether participants with other conditions were taking anti-lipid and anti-glycemic drugs, which may confound the results. Fourthly, the data was collected in 2009, and only the Chinese population was recruited; therefore, the results might not be entirely applicable to more contemporary populations and other ethnicities. Interestingly, the prevalence of dyslipidemia in this study (males: 47.7%, females: 43.9%) was similar to another Chinese study conducted in 2019 (48.27%) [58], and two multicenter Italian registries conducted between 2013 and 2016 (52.1% and 44.9%, respectively) [59]; which may potentially extend the validity of this findings. Finally, other factors, such as diet habits, may confound the relationship between sleep duration and metabolic diseases; however, the data were unavailable.

## Conclusions

The current study reveals that short sleep duration is associated with subclinical indicators of metabolic disease by using three novel indexes of TG/HDL-C, LAP, and TyG among asymptomatic adults; however, the significant associations were only observed in females. The findings provide an epidemiological basis for linking short sleep duration to an increased risk of subsequent metabolic diseases. Maintaining an appropriate sleep duration per day (7-8 h) may have significant beneficial effects to prevent the occurrence and the development of metabolic diseases.

## Supplementary information

**Additional file 1.**

## Data Availability

The datasets used in the current study are not available.

## Abbreviations

CVD: cardiovascular diseases
TG/HDL-C: triglyceride high-density lipoprotein cholesterol
HDL-C: high-density lipoprotein cholesterol
LAP: lipid accumulation product
TyG: the product of triglyceride and fasting glucose
OSA: obstructive sleep apnoea
CHNS: China health and nutrition survey
FPG: fast plasma glucose
WC: waist circumference
BMI: body mass index
hs-CRP: hypersensitive C-reactive protein
PA: physical activity
SD: standard deviation

## References

[1] Scheer FA, Hilton MF, Mantzoros CS, Shea SA (2009). Adverse metabolic and cardiovascular consequences of circadian misalignment. Proc Natl Acad Sci U S A.

[2] Lucassen EA, Rother KI, Cizza G (2012). Interacting epidemics? Sleep curtailment, insulin resistance, and obesity. Ann N Y Acad Sci.

[3] Basner M, Spaeth AM, Dinges DF (2014). Sociodemographic characteristics and waking activities and their role in the timing and duration of sleep. Sleep.

[4] Cheng GH, Chan A, Lo JC (2017). Factors of nocturnal sleep and daytime nap durations in community-dwelling elderly: a longitudinal population-based study. Int Psychogeriatr.

[5] Watson NF, Badr MS, Belenky G, Bliwise DL, Buxton OM, Buysse D, Dinges DF, Gangwisch J, Grandner MA, Kushida C (2015). Joint Consensus Statement of the American Academy of Sleep Medicine and Sleep Research Society on the Recommended Amount of Sleep for a Healthy Adult: Methodology and Discussion. Sleep.

[6] Liu Y, Wheaton AG, Chapman DP, Cunningham TJ, Lu H, Croft JB (2016). Prevalence of Healthy Sleep Duration among Adults--United States, 2014. MMWR Morb Mortal Wkly Rep.

[7] Han X, Liu B, Wang J, Pan A, Li Y, Hu H, Li X, Yang K, Yuan J, Yao P (2016). Long sleep duration and afternoon napping are associated with higher risk of incident diabetes in middle-aged and older Chinese: the Dongfeng-Tongji cohort study. Ann Med.

[8] Grandner M, Mullington JM, Hashmi SD, Redeker NS, Watson NF, Morgenthaler TI (2018). Sleep Duration and Hypertension: Analysis of > 700,000 Adults by Age and Sex. J Clin Sleep Med.

[9] Broström A, Wahlin A, Alehagen U, Ulander M, Johansson P (2018). Sex-Specific Associations Between Self-reported Sleep Duration, Cardiovascular Disease, Hypertension, and Mortality in an Elderly Population. J Cardiovasc Nurs.

[10] Austin MA, Hutter CM, Zimmern RL, Humphries SE (2004). Familial hypercholesterolemia and coronary heart disease: a HuGE association review. Am J Epidemiol.

[11] Sugiyama D, Okamura T, Watanabe M, Higashiyama A, Okuda N, Nakamura Y, Hozawa A, Kita Y, Kadota A, Murakami Y (2015). Risk of hypercholesterolemia for cardiovascular disease and the population attributable fraction in a 24-year Japanese cohort study. J Atheroscler Thromb.

[12] Fulcher J, O’Connell R, Voysey M, Emberson J, Blackwell L, Mihaylova B, Simes J, Collins R, Kirby A, Colhoun H (2015). Efficacy and safety of LDL-lowering therapy among men and women: meta-analysis of individual data from 174,000 participants in 27 randomised trials. Lancet.

[13] Nur Zati Iwani AK, Jalaludin MY, Wan Mohd Zin RM, Fuziah MZ, Hong JYH, Abqariyah Y, Mokhtar  AH, Wan Mohamud WN (2019). TG: HDL-C Ratio Is a Good Marker to Identify Children Affected by Obesity with Increased Cardiometabolic Risk and Insulin Resistance. Int J Endocrinol..

[14] Du T, Yuan G, Zhang M, Zhou X, Sun X, Yu X (2014). Clinical usefulness of lipid ratios, visceral adiposity indicators, and the triglycerides and glucose index as risk markers of insulin resistance. Cardiovasc Diabetol.

[15] Park K, Ahn CW, Lee SB, Kang S, Nam JS, Lee BK, Kim JH, Park JS (2019). Elevated TyG Index Predicts Progression of Coronary Artery Calcification. Diabetes Care.

[16] Ray L, Ravichandran K, Nanda SK (2018). Comparison of Lipid Accumulation Product Index with Body Mass Index and Waist Circumference as a Predictor of Metabolic Syndrome in Indian Population. Metab Syndr Relat Disord.

[17] Borrayo G, Basurto L, González-Escudero E, Diaz A, Vázquez A, Sánchez L, Hernández-González GO, Barrera S, Degollado JA, Córdova N, Avelar F (2018). TG/HDL-C RATIO AS CARDIO-METABOLIC BIOMARKER EVEN IN NORMAL WEIGHT WOMEN. Acta Endocrinol (Buchar).

[18] Lee YC, Park BJ, Lee JH (2021). Sex Differences in the Relationship Between High-Risk Drinking and the Triglyceride-Glucose (TyG) Index: An Analysis Using 2013 and 2015 Korean National Health and Nutrition Examination Survey Data. Alcohol Alcohol.

[19] Zhang Y, Hu J, Li Z, Li T, Chen M, Wu L, Liu W, Han H, Yao R, Fu L (2019). A Novel Indicator Of Lipid Accumulation Product Associated With Metabolic Syndrome In Chinese Children And Adolescents. Diabetes Metab Syndr Obes.

[20] Zou J, Wang Y, Xu H, Xia Y, Qian Y, Zou J, Guan J, Chen B, Yi H, Yin S (2020). The use of visceral adiposity variables in the prediction of obstructive sleep apnea: evidence from a large cross-sectional study. Sleep Breath.

[21] Dong L, Lin M, Wang W, Ma D, Chen Y, Su W, Chen Z, Wang S, Li X, Li Z, Liu C (2020). Lipid accumulation product (LAP) was independently associatedwith obstructive sleep apnea in patients with type 2 diabetes mellitus. BMC Endocr Disord.

[22] Bikov A, Frent SM, Meszaros M, Kunos L, Mathioudakis AG, Negru AG, Gaita L, Mihaicuta S (2021). Triglyceride-Glucose Index in Non-Diabetic, Non-Obese Patients with Obstructive Sleep Apnoea. J Clin Med.

[23] Bikov A, Meszaros M, Kunos L, Negru AG, Frent SM, Mihaicuta S (2021). Atherogenic Index of Plasma in Obstructive Sleep Apnoea. J Clin Med.

[24] Kim JH, Jung DH, Kwon YJ, Lee JI, Shim JY (2019). The impact of the sleep duration on NAFLD score in Korean middle-aged adults: a community-based cohort study. Sleep Med.

[25] Regitz-Zagrosek V, Lehmkuhl E, Mahmoodzadeh S (2007). Gender aspects of the role of the metabolic syndrome as a risk factor for cardiovascular disease. Gend Med.

[26] Ye X, Yu Z, Li H, Franco OH, Liu Y, Lin X (2007). Distributions of C-reactive protein and its association with metabolic syndrome in middle-aged and older Chinese people. J Am Coll Cardiol.

[27] Lee YC, Park BJ, Lee JH (2021). Sex Differences in the Relationship Between High-Risk Drinking and the Triglyceride-Glucose (TyG) Index: An Analysis Using 2013 and 2015 Korean National Health and Nutrition Examination Survey Data. Alcohol Alcohol.

[28] Ainsworth BE, Haskell WL, Whitt MC, Irwin ML, Swartz AM, Strath SJ, O’Brien WL, Bassett DR, Schmitz KH, Emplaincourt PO (2000). Compendium of physical activities: an update of activity codes and MET intensities. Med Sci Sports Exerc.

[29] Wang S, Xu L, Jonas JB, You QS, Wang YX, Yang H (2011). Prevalence and associated factors of dyslipidemia in the adult Chinese population. PLoS One.

[30] Opoku S, Gan Y, Yobo EA, Tenkorang-Twum D, Yue W, Wang Z, Lu Z (2021). Awareness, treatment, control, and determinants of dyslipidemia among adults in China. Sci Rep.

[31] Greenland S (1989). Modeling and variable selection in epidemiologic analysis. Am J Public Health.

[32] Theorell-Haglöw J, Lindberg E (2016). Sleep Duration and Obesity in Adults: What Are the Connections?. Curr Obes Rep.

[33] Song Q, Liu X, Zhou W, Wu S, Wang X (2020). Night sleep duration and risk of each lipid profile abnormality in a Chinese population: a prospective cohort study. Lipids Health Dis.

[34] Kim JY, Yadav D, Ahn SV, Koh SB, Park JT, Yoon J, Yoo BS, Lee SH (2015). A prospective study of total sleep duration and incident metabolic syndrome: the ARIRANG study. Sleep Med.

[35] Bos MM, Noordam R, van den Berg R, de Mutsert R, Rosendaal FR, Blauw GJ, Rensen PCN, Biermasz NR, van Heemst D (2019). Associations of sleep duration and quality with serum and hepatic lipids: The Netherlands Epidemiology of Obesity Study. J Sleep Res.

[36] Kahn HS (2005). The “lipid accumulation product” performs better than the body mass index for recognizing cardiovascular risk: a population-based comparison. BMC Cardiovasc Disord.

[37] Taverna MJ, Martínez-Larrad MT, Frechtel GD, Serrano-Ríos M (2011). Lipid accumulation product: a powerful marker of metabolic syndrome in healthy population. Eur J Endocrinol.

[38] Guo SX, Zhang XH, Zhang JY, He J, Yan YZ, Ma JL, Ma RL, Guo H, Mu LT, Li SG (2016). Visceral Adiposity and Anthropometric Indicators as Screening Tools of Metabolic Syndrome among Low Income Rural Adults in Xinjiang. Sci Rep.

[39] Navarro-González D, Sánchez-Íñigo L, Pastrana-Delgado J, Fernández-Montero A, Martinez JA (2016). Triglyceride-glucose index (TyG index) in comparison with fasting plasma glucose improved diabetes prediction in patients with normal fasting glucose: The Vascular-Metabolic CUN cohort. Prev Med.

[40] Feng X, Liu Q, Li Y, Zhao F, Chang H, Lyu J (2019). Longitudinal study of the relationship between sleep duration and hypertension in Chinese adult residents (CHNS 2004-2011). Sleep Med.

[41] Itani O, Jike M, Watanabe N, Kaneita Y (2017). Short sleep duration and health outcomes: a systematic review, meta-analysis, and meta-regression. Sleep Med.

[42] Stern JH, Grant AS, Thomson CA, Tinker L, Hale L, Brennan KM, Woods NF, Chen Z (2014). Short sleep duration is associated with decreased serum leptin, increased energy intake and decreased diet quality in postmenopausal women. Obesity (Silver Spring).

[43] Broussard JL, Kilkus JM, Delebecque F, Abraham V, Day A, Whitmore HR, Tasali E (2016). Elevated ghrelin predicts food intake during experimental sleep restriction. Obesity (Silver Spring).

[44] Cui H, López M, Rahmouni K (2017). The cellular and molecular bases of leptin and ghrelin resistance in obesity. Nat Rev Endocrinol.

[45] Chou YT, Li CH, Shen WC, Yang YC, Lu FH, Wu JS, Chang CJ (2020). Association of sleep quality and sleep duration with serum uric acid levels in adults. PLoS One.

[46] Wu AH, Gladden JD, Ahmed M, Ahmed A, Filippatos G (2016). Relation of serum uric acid to cardiovascular disease. Int J Cardiol.

[47] Solarz DE, Mullington JM, Meier-Ewert HK (2012). Sleep, inflammation and cardiovascular disease. Front Biosci (Elite Ed).

[48] Besedovsky L, Lange T, Haack M (2019). The Sleep-Immune Crosstalk in Health and Disease. Physiol Rev.

[49] Calvin AD, Covassin N, Kremers WK, Adachi T, Macedo P, Albuquerque FN, Bukartyk J, Davison DE, Levine JA, Singh P (2014). Experimental sleep restriction causes endothelial dysfunction in healthy humans. J Am Heart Assoc.

[50] Incalza MA, D’Oria R, Natalicchio A, Perrini S, Laviola L, Giorgino F (2018). Oxidative stress and reactive oxygen species in endothelial dysfunction associated with cardiovascular and metabolic diseases. Vascul Pharmacol.

[51] Rolls A, Schaich Borg J, de Lecea L (2010). Sleep and metabolism: role of hypothalamic neuronal circuitry. Best Pract Res Clin Endocrinol Metab.

[52] Mosca L, Barrett-Connor E, Wenger NK (2011). Sex/gender differences in cardiovascular disease prevention: what a difference a decade makes. Circulation.

[53] Rossi P, Francès Y, Kingwell BA, Ahimastos AA (2011). Gender differences in artery wall biomechanical properties throughout life. J Hypertens.

[54] Miller MA, Kandala NB, Kivimaki M, Kumari M, Brunner EJ, Lowe GD, Marmot MG, Cappuccio FP (2009). Gender differences in the cross-sectional relationships between sleep duration and markers of inflammation: Whitehall II study. Sleep..

[55] Lauderdale DS, Knutson KL, Yan LL, Liu K, Rathouz PJ (2008). Self-reported and measured sleep duration: how similar are they?. Epidemiology.

[56] Wang D, Chen J, Zhou Y, Ma J, Zhou M, Xiao L, He M, Zhang X, Guo H, Yuan J, Chen W (2019). Association between sleep duration, sleep quality and hyperlipidemia in middle-aged and older Chinese: The Dongfeng-Tongji Cohort Study. Eur J Prev Cardiol.

[57] Barros D, García-Río F. Obstructive sleep apnea and dyslipidemia: from animal models to clinical evidence. Sleep. 2019;42(3):zsy236. 10.1093/sleep/zsy236 30476296

[58] Gao H, Wang H, Shan G, Liu R, Chen H, Sun S, Liu Y (2021). Prevalence of dyslipidemia and associated risk factors among adult residents of Shenmu City, China. PLoS One.

[59] Calabrò P, Gragnano F, Di Maio M, Patti G, Antonucci E, Cirillo P, Gresele P, Palareti G, Pengo V, Pignatelli P (2018). Epidemiology and Management of Patients With Acute Coronary Syndromes in Contemporary Real-World Practice: Evolving Trends From the EYESHOT Study to the START-ANTIPLATELET Registry. Angiology.

