# Supplementary material for: Sex-specific association of sleep duration with subclinical indicators of metabolic diseases among asymptomatic adults
Source: Lipids Health Dis. 2022 Jan 23;21:16. doi: 10.1186/s12944-022-01626-w (PMC8783994; doi:10.1186/s12944-022-01626-w)
Supplement: Supplementary file 1 — Additional file 1. [file 12944_2022_1626_MOESM1_ESM.docx]

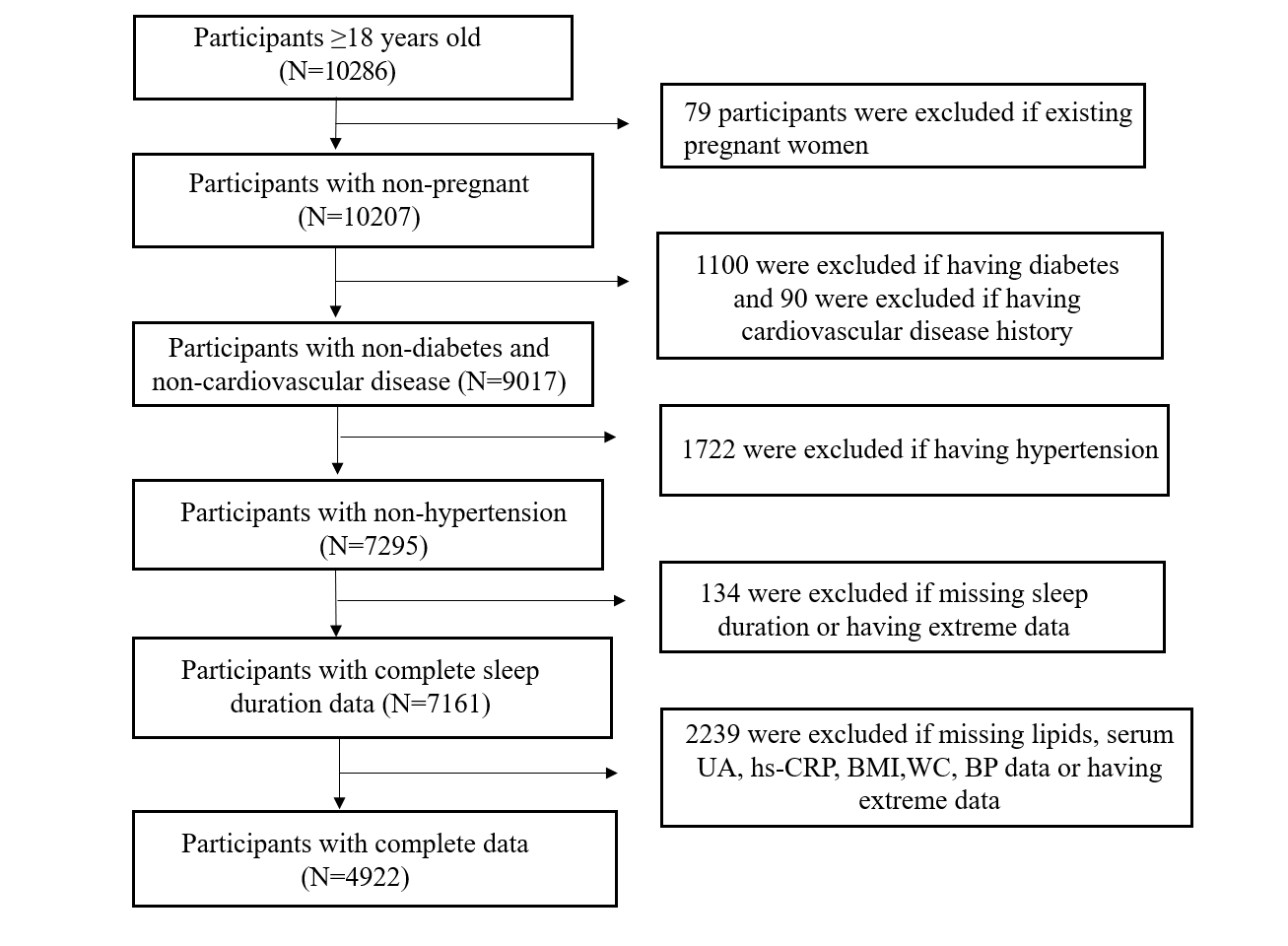


**Figure S1 Sampling flowchart and participants enrollment**

| **Table S1** **Characteristics between included participants and those excluded，mean (SD) or n (%)** | | | |
| --- | --- | --- | --- |
|  | **Included (n=4922)** | **Excluded (n=5364)** | ***P*** |
| Age (years) | 46.60 (14.22) | 52.24 (16.39) | <0.001 |
| BMI (kg/m2) | 22.65 (3.10) | 24.07 (3.70) | <0.001 |
| WC (cm) | 80.23 (9.40) | 85.38 (10.73) | <0.001 |
| SBP (mmHg) | 116.68 (12.25) | 132.81 (21.05) | <0.001 |
| DBP (mmHg) | 76.01 (8.20) | 84.63 (12.32) | <0.001 |
| Sex |  |  |  |
| Male | 2194 (44.6) | 2741 (51.1) | <0.001 |
| Female | 2728 (55.4) | 2623 (48.9) |  |
| Region |  |  |  |
| North | 2689 (54.6) | 2991 (55.8) | <0.001 |
| South | 2233 (45.4) | 2373 (44.2) |  |
| Area of city |  |  |  |
| Urban | 1510 (30.7) | 1998 (37.2) | <0.001 |
| Rural | 3412 (69.3) | 3366 (62.8) |  |
| Education |  |  |  |
| Less than primary | 1913 (38.9) | 2132 (39.8) |  |
| Middle-high school | 2381 (48.4) | 2503 (46.7) |  |
| Tech and more | 625 (12.7) | 727 (13.6) |  |
| Income (yuan) |  |  |  |
| Low | 1640 (34.1) | 1716 (33.0) |  |
| Medium | 1637 (34.1) | 1733 (32.3) |  |
| High | 1528 (31.8) | 1746 (32.6) |  |
| Sedentary time (hours) | 2.85 (2.32) | 2.76 (2.38) |  |
| Smoke |  |  |  |
| Yes | 1475 (30.0) | 1685 (31.4) | 0.004 |
| No | 3445 (70.0) | 3473 (67.3) |  |
| Drank alcohol |  |  |  |
| Yes | 1561 (31.7) | 1791 (34.7) | 0.001 |
| No | 3360 (68.3) | 3367 (65.3) |  |
| Sleep duration (hours) | 7.98 (1.12) | 7.97 (1.40) |  |
| <7 | 394 (8.0) | 596 (11.8) | <0.001 |
| 7-9 | 3436 (69.8) | 3163 (62.9) |  |
| ≥9 | 1092 (22.2) | 1272 (25.3) |  |

BMI: body mass index; WC: waist circumference; SBP: systolic blood pressure; DBP: diastolic blood pressure
